# Cryo-EM Studies of Genome Organization and Transcription Complexes

**DOI:** 10.1063/4.0000865

**Published:** 2025-10-27

**Authors:** Seychelle M Vos

**Affiliations:** Massachusetts Institute of Technology, Cambridge, Massachusetts, USA

## Abstract

RNA polymerase (Pol) II is regulated during all stages of transcription to ensure appropriate gene expression. This regulation involves interaction with various factors that modulate Pol II activity as it traverses across genes. I will discuss recent cryo-electron microscopy(EM) work that has uncovered how Pol II is both negatively and positively regulated by transcription elongation factors. Pol II activity is also influenced by the organization of the genome. Genome organisation is regulated at multiple levels ranging from the underlying DNA sequence to large scale interactions between chromosomes. Our recent efforts to understand how these multiple levels of genome organisation are used to regulate gene expression will be discussed.

